# Quality and Diagnosis on the Lateral View of Pediatric Upper Gastro-Intestinal Series

**DOI:** 10.3390/children11020151

**Published:** 2024-01-24

**Authors:** Shyam Sunder B. Venkatakrishna, Mohamed Elsingergy, Juan S. Calle-Toro, Rebecca Dennis, Hansel J. Otero, Savvas Andronikou

**Affiliations:** 1 Department of Radiology, Children’s Hospital of Philadelphia, Philadelphia, PA 19104, USA; 2 Department of Radiology, University of Texas Health Science Center at San Antonio, San Antonio, TX 78229, USA; 3 Department of Radiology, Perelman School of Medicine, University of Pennsylvania, Philadelphia, PA 19104, USA

**Keywords:** UGIS, lateral view, descending staircase sign, duodenum redundum, malrotation

## Abstract

Background: The standard imaging technique for the diagnosis of intestinal malrotation remains the upper gastro-intestinal series (UGIS). The lateral view is promoted as important for making a diagnosis. For this, the lateral view should be of adequate quality, and radiologists must know the normal appearance as well as the appearance of duodenal variants, as misdiagnosis may lead to unnecessary surgery. Objective: We aimed to evaluate the quality, findings including the prevalence of the ”descending staircase” configuration and its correspondence to a diagnosis of duodenum redundum. Materials and Methods: This was a retrospective study and was conducted in a large tertiary children’s hospital in the United States. A retrospective review of UGI fluoroscopy exams in children aged ≤ 18 years between January and December 2018 was performed by a pediatric radiologist. First, the lateral view images/cine-loops were assessed independently, followed by the anteroposterior (AP) view. The studies which were designated to have an adequate lateral view were evaluated for configuration of the duodenum and recorded as: normal, abnormal, or normal variant. Also, the presence of a descending staircase configuration was correlated with an AP view for a diagnosis of duodenum redundum. Results: A total of 26 children (26%) (males:16; females:10) with age range 0 to 16 years had adequate lateral views during UGI exams for inclusion. Of the 26, 18 (69%) were reported as normal, 7 (27%) were reported as having a descending staircase and 1 (4%) was reported as abnormal. The AP view demonstrated 2 abnormal studies (1 malrotation and 1 non-rotation), 6 duodenum redundum and 18 normal exams. The one abnormal lateral duodenum was confirmed as a non-rotation on AP view; the second patient with an abnormal AP view had a normal appearance on the lateral view. Conclusions: A total of 26% of UGI studies had adequate lateral views for interpretation. Of these, nearly a quarter (23%) demonstrated the descending stair-case sign corresponding to a diagnosis of duodenum redundum on the AP view. If the lateral view had been used alone, there would have been a missed diagnosis in one patient.

## 1. Introduction

Despite efforts to move to an ultrasound diagnosis, the standard imaging technique for the diagnosis of malrotation and midgut volvulus in newborns with bilious vomiting remains the fluoroscopically guided upper gastro-intestinal series (UGIS) [1,2,3,4,5,6]. Malrotation is a congenital condition associated with abnormal bowel rotation and can include both small and large intestines. The anomalies can be wide-ranging, from asymptomatic non-rotation to easily diagnosed omphalocele. It is an important condition that needs to be diagnosed on imaging, especially in children presenting to the emergency setting with bilious vomiting, as malrotation predisposes to midgut volvulus with bowel ischemia and potentially significant morbidity and mortality if untreated.

Children are referred to radiologists for demonstration of the duodenal anatomy and diagnosis of malrotation and mid-gut volvulus. The radiologist’s interpretation directly impacts management—patients with normal anatomy are often discharged while those with abnormal duodenal C-loops undergo surgery, sometimes as an emergency [7]. The configuration of the duodenum and position of the duodenojejunal (DJ) flexure on an antero-posterior (frontal) view of the UGIS are most commonly used for diagnosis. Exclusion of malrotation on the UGIS depends on the demonstration of the duodeno-jejunal (DJ) flexure in the correct anatomical position in the left upper quadrant of the abdomen. A diagnosis of malrotation is therefore made if the DJ-flexure is in an abnormal position: (a) overlying or to the right of the left pedicle of 1st lumbar vertebrae; (b) at a more caudal position than the first part of duodenum [8]. A total of 75% of patients with malrotation have one or more radiologic signs, while up to 25% of patients with malrotation have no signs and some reports indicate a false-positive rate up to 15% [8,9].

When performing the UGIS, the radiologist follows a bolus of contrast (the first pass) moving through the duodenum (duodenal C-loop) fluoroscopically [8]. Radiologists routinely place the patient in the right-side-down lateral position to allow gravity to aid a contrast passage from the stomach into the duodenum. However, to achieve the diagnostic image for meeting the above anatomic criteria for excluding or making a diagnosis of malrotation, the patient has to be repositioned into the anteroposterior (AP) view (frontal view)—a maneuver known as the “duodenal roll”, which more often than not results in patient mal-positioning for the most important component of the study. This has been shown to be a common cause for potential false–positive results [7].

**Figure 1:**
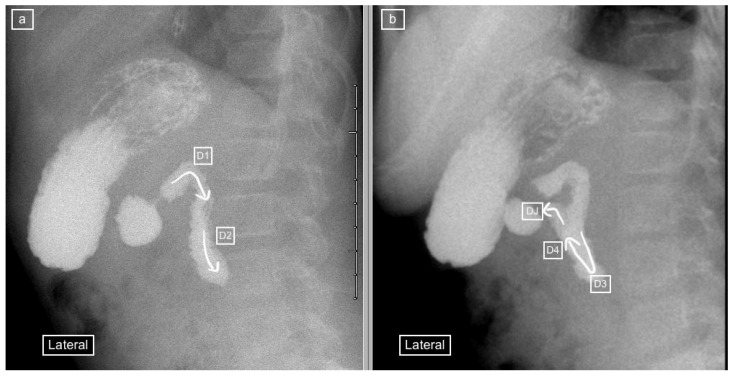
(**a**) early and (**b**) later view: Sequential lateral views from an upper gastrointestinal tract study (UGIS) in an 8-year-old boy demonstrating the normal configuration of the duodenum which courses posteriorly (retroperitoneal) from the antrum (D1), descends posteriorly (D2), remains posterior at its most inferior portion (D3), ascends posteriorly again through (D4) and only then courses anteriorly from the duodeno-jejunal (DJ) flexure at the same height as the antrum and D1.

Radiologists such as Koplewitz and Daneman have recommended the use of the lateral view for diagnosis, with a posterior retroperitoneal position of the duodenal loop and DJ flexure at the level of the gastric antrum and duodenal cap representing a normal appearance [10], (Figure 1). The lateral position not only allows distinguishing an abnormal anterior from a normal posterior retroperitoneal position of the duodenum but also avoids having to rotate the patient during the study, thereby simplifying the procedure and avoiding missing the important component of the study. However, for the lateral view to be useful, it must be of adequate quality. The quality of the lateral view is often sacrificed in favor of achieving an adequate frontal view during the highly dynamic ”duodenal roll” maneuver. Time is of essence for viewing and capturing the duodenal anatomy because delay in performing the ”duodenal roll” for obtaining the frontal view can lead to contrast flowing into the jejunum with the overlapping intestinal loops affecting the quality and increasing the potential for misdiagnosis [7].

In addition, the presence of duodenal normal variants such as *duodenum redundum, may result in* misdiagnosis and lead to unnecessary surgery. Duodenum redundum (“redundant duodenum”) and duodenum inversum are the two known normal variants of the duodenum [8,11,12,13]. Duodenum redundum is a normal variant with an elongated third portion of the duodenum resulting in a ”W” or ”WV” configuration of the frontal view. There are no reports of the appearance of duodenum redundum on the lateral view, however, or whether these may mimic pathology. We have anecdotally noted a distinctive ”descending staircase” pattern in cases of duodenum inversum on the lateral view, which deviates from the normal and may be diagnostic but may also be confusing for interpretation of the lateral view.

In this study, we aimed to evaluate the quality and findings on the lateral view of UGIS, including the prevalence of the ”descending staircase” configuration and its correspondence to a diagnosis of duodenum redundum on the frontal view, in a cohort of children imaged with UGIS for suspected malrotation/midgut volvulus.

## 2. Materials and Methods

This was a retrospective Institutional Review Board (IRB) approved study, compliant with the Health Insurance Portability and Accountability Act (HIPAA), and was conducted in a large tertiary children’s hospital in the United States. A retrospective review of UGI fluoroscopy exams in children aged ≤ 18 years was performed between January and December 2018 by a pediatric radiologist with 24 years of experience in a variety of settings. There was exclusion of patients from the study if they had a history of previous gastrointestinal (GI) surgery (such as Ladd’s procedure) or if they did not have a follow-up clinical examination for confirmation of outcome: (a) surgery for malrotation/volvulus at first presentation or follow-up presentation (classified as abnormal malrotation) or (b) discharge without re-presentation (classified as normal).

**Figure 2:**
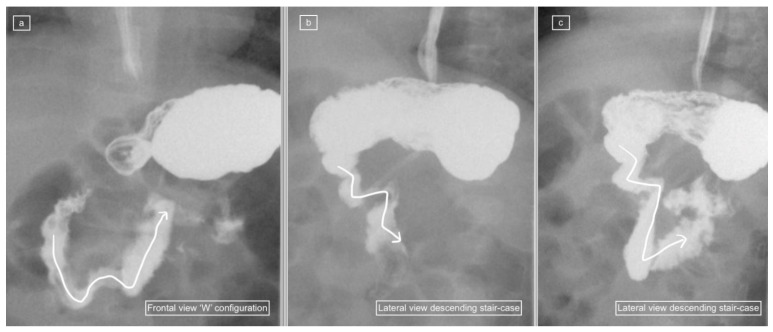
(**a**–**c**): Representative images captured during an UGIS in a 3-month-old boy demonstrating duodenum redundum on the frontal and lateral views. (**a**) Frontal view demonstrates redundancy of the duodenal loop which instead of having a C-loop configuration now has a W-loop configuration (white line) and a normal position of the duodeno-jejunal flexure (immediately distal to the end of the arrowhead). (**b**) Early and (**c**) later lateral views captured prior to the frontal view in (**a**) demonstrate a descending staircase configuration of D1 and D2 oriented posteriorly, after which the duodenum ascends in the retroperitoneal position, leading up to the duodeno-jejunal flexure in the expected normal position at the same height as D1, before coursing anteriorly.

**Figure 3:**
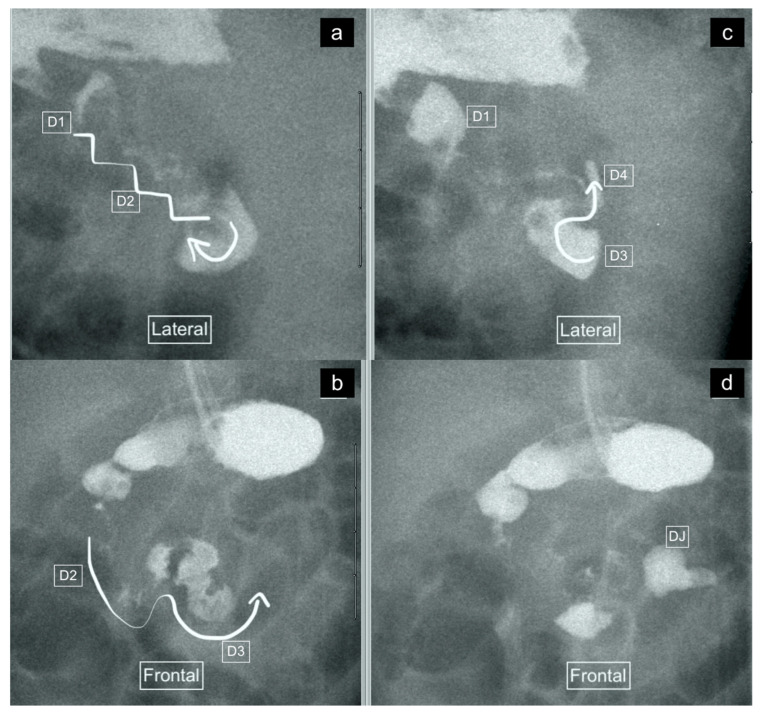
(**a**–**d**): Representative images captured during an UGIS in a 49-day-old boy demonstrating duodenum redundum on the frontal and lateral views, with the order of images representing the order that the images were captured through multiple repositioning maneuvers. (**a**) Lateral view demonstrating the descending staircase sign oriented posteriorly from D1 and involving D2. (**b**) Frontal view demonstrating the W-loop configuration of duodenum redundum made up here by D2 and D3. (**c**) Repositioning in the lateral view demonstrates the posterior position of D3 inferiorly and that the ascending D4 remains posterior. (**d**) Return to the frontal position now demonstrates the contrast outlining the duodeno-jejunal flexure (DJ) just slightly inferior to D1 and to the left of the spine—considered within the normal range allowing for patient positioning.

**Figure 4:**
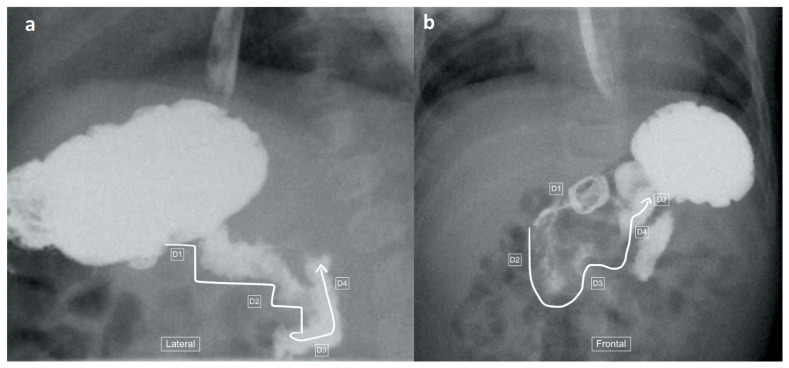
(**a**,**b**): Representative images captured during an UGIS in a 2-month-old boy demonstrating duodenum redundum on the lateral and frontal views. (**a**) Lateral view demonstrates a descending staircase configuration of D1 and D2 oriented posteriorly, after which the duodenum remains (D3) and ascends (D4) in the retroperitoneal position, leading towards the duodeno-jejunal junction (not shown here). (**b**) Frontal view demonstrates redundancy of the duodenal loop which, instead of having a C-loop configuration, has a W-loop configuration, made up of D2, D3 and D4 (white line) and a normal position of the duodeno-jejunal flexure (DJ).

The recorded images of the lateral view or cine loops, including the lateral position, were reviewed independently before a review of findings on the frontal (AP) view. Studies were categorized as having ”not captured” or ”inadequately captured” (available but uninterpretable due to inadequate luminal contrast outlining the duodenum on the view) images of the lateral view. We calculated the proportion of studies with adequate lateral views (by excluding the non-captured and inadequately captured cases), and only studies designated to have an adequate lateral view were evaluated further regarding the configuration of the duodenum. This latter subset was then categorized as ”normal”, ”descending staircase”, or abnormal from the lateral view. The descending staircase sign was defined as a stepwise inferior descent of the duodenal course from D1 through D2 oriented posteriorly, after which D3 and D4 ascend in the retroperitoneal position, leading up to the duodeno-jejunal flexure at the same height as D1, before coursing anteriorly (Figure 2, Figure 3 and Figure 4).

The final diagnosis was determined from the frontal view as the standard, and the ”descending staircase” configuration was specifically compared with the AP view for the diagnosis of duodenum redundum.

### Imaging Technique

The standard UGI exams in our institution are performed by having the child swallow contrast (usually thin barium), using any one of four methods of administration: bottle, cup, syringe in the mouth or via a nasogastric tube. We avoid overfilling the stomach because a distended stomach can obscure the DJ flexure. We begin with the right-side-down position because gravity can assist with duodenal filling—this position is also used for viewing and recording the lateral duodenal view and identifying a normal posterior and retroperitoneal position of the duodenum. Most radiologists and surgeons require a frontal view for diagnosis, and therefore the child is now required to turn into a supine position on the bed which must be performed rapidly (the ”duodenal roll”) to avoid obscuring important anatomy by contrast flowing into overlying loops of jejunum. We use pulsed fluoroscopy and the recording of a cine loop during the ”duodenal roll”. This is the crucial step of capturing the duodenal “C-loop” during a first pass without overlying contrast in the jejunum. The UGIS in our study were performed by multiple radiologist operators (there were more than 30 pediatric radiologists in our department during the period of the study) who while following this protocol also used individual components to their technique. These diagnostic UGIS reports form the initial clinical diagnostic interpretation for this paper. This technique has been previously published by our group [8].

## 3. Results

After application of inclusion and exclusion criteria, a convenience sample of 100 studies was selected. Of these, 26 (26%) patients’ UGI exams were designated as having an adequate lateral view for evaluation, (male:16; female:10) with age range of 0 to 16 years (mean age ± SD: 2.8 ± 4.7 years).

Reasons for referral for these 26 studies were: Vomiting reflux—18, Pre-surgical evaluation of duodenal anatomical location—3, Emergency for bilious emesis—1, Congenital diaphragmatic hernia—1, Dysphagia—1, concern for anatomic small bowel obstruction—1, Fussiness—1.

The routes of contrast administration were: Traditional oral route (i.e., contrast swallow)—22, and via nasogastric tube—4.

**Figure 5:**
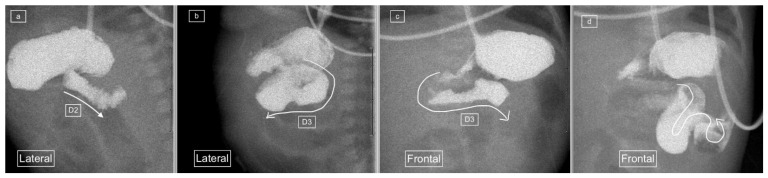
(**a**–**d**): UGIS showing an abnormal duodenum in a 12-day-old boy with non-rotation. (**a**) Lateral view demonstrates a “slope-like” descent of D2 oriented posteriorly. (**b**) D3 courses anteriorly from this point. (**c**,**d**) represent sequential image captures in the frontal view which show the horizontally oriented D3 with an inferior descent rather than a superior ascent to D4. Most of the small bowel was positioned on the right on follow-up images in keeping with non-rotation rather than malrotation.

A total of 18 of the 26 adequate lateral UGI exams (69%) were interpreted as normal, 7/26 (27%) were interpreted as demonstrating the descending staircase configuration (Figure 2, Figure 3 and Figure 4) and 1/26 (4%) was interpreted as abnormal (Figure 5).

**Figure 6:**
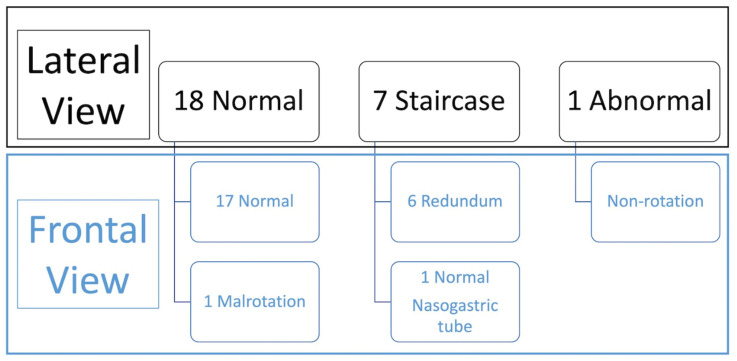
Diagram demonstrating the findings on the lateral view against the findings on the frontal view, with the frontal view representing the standard for this study.

Comparison with the AP view was performed (summarized in Figure 6) which demonstrated 2 abnormal exams (1 malrotation and 1 non-rotation), 6 duodenum redundum and 18 normal studies.

**Figure 7:**
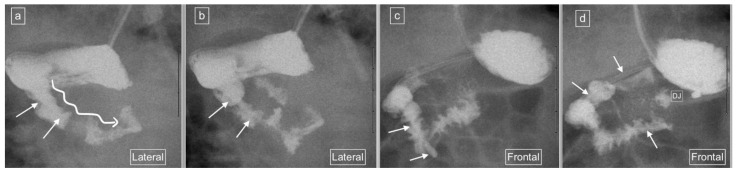
Representative images captured during an UGIS in a 6-month-old girl showing an indwelling enteric tube with its tip terminating in the third part of the duodenum. (**a**) Early and (**b**) later lateral views captured, demonstrate what was interpreted as a descending staircase sign (long winding arrow) but actually represents distortion of the C-loop configuration with duodenal spasm due to the presence of the enteric tube (short arrows). (**c**,**d**) are representative frontal view image captures with a relatively normal C-loop configuration and a normal position of the duodeno-jejunal flexure (DJ). The enteric tube is seen within the duodenum (arrows).

**Figure 8:**
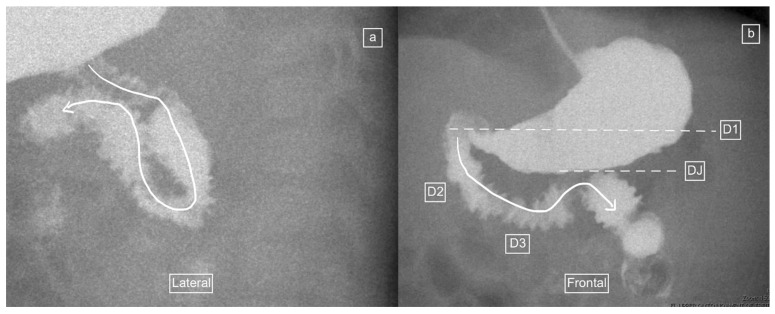
Upper-gastrointestinal tract series in a 159-day-old girl with a false–negative lateral view and a diagnosis of malrotation on the frontal view. (**a**) Lateral view demonstrates what appears to be a normal course of the duodenum (winding arrow) into its posterior retroperitoneal position, with a presumed normal position of the duodenojejunal flexure, before the jejunum takes and anterior course. (**b**) Frontal view, considered the standard for this study, demonstrates the duodenal loop (from D1 through D2, D3 and D4 denoted by the winding arrow) and a caudal (low) position of the duodeno-jejunal flexure (DJ) in comparison to D1 (the positional differences denoted by the dashed lines), consistent with malrotation.

Six of the seven lateral views with descending staircase duodenums had duodenum redundum on the AP view and one was reported as normal, but on review contained a naso-jejunal tube in situ, causing distortion of the duodenal anatomy and spasm of the bowel (Figure 7). The single abnormal lateral duodenum was confirmed on AP as a non-rotation (Figure 5). There was one patient with a normal lateral view but malrotation on the frontal view (Figure 8).

## 4. Discussion

The gold standard imaging examination for the diagnosis of rotational disorders of the midgut has been the UGIS. However, there are several challenges associated with performing UGI examinations reported in the literature [14,15]. These factors can affect the quality of the scan, and include the randomness of the boluses advancing into the duodenum and the repositioning required to obtain an AP view for a diagnosis [14,15]. Exams considered of inadequate quality can lead to non-visualization of the duodenum and DJ flexure, leading to errors in diagnosis. This is because demonstration of DJ flexure in its normal anatomical position is considered essential for exclusion of malrotation on UGI studies [15,16].

The role of the lateral view as a useful adjunct for diagnosing malrotation has previously been published by Koplewitz and Daneman [10]. These authors reported the findings of UGIS in 49 children, out of which 40 had lateral views, and the course of the duodenum on the lateral view could be followed in 27 of these patients, while in 13 patients it was inconclusive [10]. In total, therefore, only 27 of 49 (55%) studies had available and adequate lateral views in the Koplewitz study. Our study had an even lower proportion of 26% of adequate lateral views in UGIS for evaluation. This likely reflects the primary goal of the UGIS for most radiologists being the recording of an adequate frontal view. Despite having only just over half of cases with diagnostic lateral views, Koplewitz and Daneman reported that the lateral view can be a useful adjunct for diagnosis, especially when there is an inconclusive AP view [10]. However, Sizemore et al. reported three cases of false negative (normal) lateral views in patients with malrotation [15] and our own findings include 1 of 26 patients who demonstrated a normal lateral view but had malrotation on the frontal view (Figure 8). These isolated false negative cases suggest that the lateral view should be used with some caution and should not be used in isolation but rather in conjunction with the frontal view, to avoid unnecessary surgery.

Despite the relatively frequent occurrence of duodenal redundancy reported (29% in the study by Sizemore et al. [15]), neither these authors nor any of the other papers reporting and promoting the use of the lateral view refer to the appearance of duodenal variants on lateral views, and whether these cause diagnostic dilemmas such as false positive diagnoses of malrotation. Clinical radiologists should be able to recognize normal variations in order to avoid overdiagnosis of malrotation, resulting in unwanted surgery. We demonstrated a frequency of 23% duodenal variants in our patient population and all of these (100%) demonstrated a characteristic descending staircase sign on the lateral view. However, one case designated as having a descending staircase sign on the lateral view showed a normal duodenum on the frontal view and on review, there is a clear distortion of the duodenum caused by an indwelling naso-jejunal tube with spasm of the bowel. This pitfall is a known potential diagnostic pitfall on the frontal view, which causes malposition of the DJ flexure but is not well documented in the lateral view.

The likely reason that most radiologists do not like to delay the duodenal roll in order to acquire an adequate lateral view is that the delay risks compromising the AP view—reflected by the low rate of adequate lateral views—26% in our sample and 55% in the Koplewitz et al. study [10]. A combination of the lateral view with an AP view can be complementary in supporting the correct diagnosis, with avoidance of ”false positives” and unnecessary surgery. As Long et al. have concluded previously for the frontal view and now shown again for the lateral view, false positives can be avoided by recognizing the variants of normal duodenum [17]. In addition to our recommendation of using the lateral view in conjunction with an AP view for diagnosis, we also recommend (a) an assessment of the lateral view quality before making conclusions, (b) caution when there are indwelling enteric tubes and (c) awareness of the descending staircase sign of duodenum redundum, which represents a variation of normal rather than pathology.

Other imaging techniques for diagnosing malrotation and midgut volvulus include ultrasound, CT and MRI [18,19,20,21], where demonstration of a retroperitoneal duodenum passing the deep [posterior] to the superior mesenteric artery and vein is considered the normal anatomic arrangement. These diagnostic imaging modalities have not gained widespread acceptance. Ultrasound is an obvious choice because of its low cost and lack of ionizing radiation, making it attractive for use in children, as well as its portability, allowing it to be used in the emergency room, in clinics and at the bedside. However, identifying the duodenum passing deep to the superior mesenteric artery (SMA)/superior mesenteric vein (SMV) on ultrasound is challenging or even impossible in some patients due to obscuring bowel gas in the stomach or other portions of the bowel [18]. MRI which also does not use ionizing radiation, may require anesthesia for children under six years of age, which increases the risk, causes delays and is often inaccessible during after-hours when these emergencies present. Using CT to identify the duodenal anatomy has been tested and can be achieved with high reliability, but even though CT scanners are widely available and radiologists of all levels have good expertise with CT, there is a reluctance to apply CT scans in children, due to the perceived risks of radiation [18]. The UGIS therefore remains the mainstay for diagnosing or excluding malrotation and midgut volvulus in children, and any mechanisms for improving the diagnostic yield, such as the use of the lateral view, should be entertained seriously. Recognition of variations of normal on the lateral view therefore becomes more important for the radiologist.

## 5. Limitations

This is a retrospective study with a small sample size. The imaging reviewed for this study was from DICOM files on PACS, and not from the live fluoroscopy exams, which precludes evaluation of what was seen but not recorded. In addition, there were no other imaging modalities used for comparison with the UGIS exams and patient outcome is assumed to reflect the ground truth where, in fact, patients with malrotation may survive without re-presenting.

## 6. Conclusions

A total of 26% of UGI studies had lateral views of adequate quality for interpretation. Of these, nearly a quarter (23%) demonstrated the descending staircase sign corresponding to a diagnosis of duodenum redundum on the AP view. Lateral views of the duodenum on UGIS can indeed be useful but as in one of our cases, can also yield false negative results and should be used in conjunction with the frontal view. Obtaining both adequate lateral and frontal views of the duodenum on the first pass presents a challenge, as delay in turning the patient to obtain an adequate lateral view risks compromising the frontal view. Alternative imaging for assessment of the duodenal anatomy should be entertained. However, while the UGIS remains the diagnostic standard, radiologists should be aware of variant duodenum configurations including the descending staircase sign, described in this paper, to avoid misdiagnosis and unnecessary surgery.

## Data Availability

The data presented in this study are available on reasonable request from the corresponding author. The data are not publicly available due to privacy restrictions or ethical restrictions.
